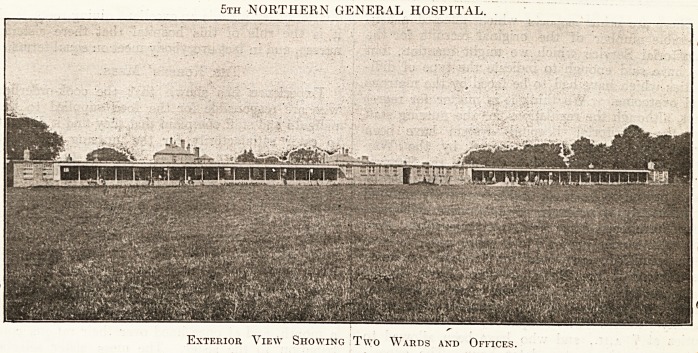# The Nursing System: Some Difficulties and Differences

**Published:** 1915-10-23

**Authors:** 


5th NORTHERN GENERAL HOSPITAL.

Exterior View Showing Two Wards and Offices.

### THE TERRITORIAL HOSPITAL.

### II.—The Nursing System: Some Difficulties and Differences.

In some ways, and in many places, the general efficiency of the Territorial Force Nursing Service as encountered in a Territorial hospital is unexpectedly efficient. On its inception it was decided to invite nurses willing to join this force to commit and bind themselves to conditions which made its foundations insecure. Each nurse had to register her name, and to guarantee that in the event of war her services should be at once available and at the command of the Service. Once accepted, she was bound by her guarantee. No matter what high position she might be occupying, or what promotion she had gained by devotion and ability, she was liable to relinquish it and join for war service. When the Territorial Nursing Service was first created this plan was severely criticised, and properly, because it placed every nurse who was accepted to serve in a wrong position; for no woman, or, for that matter, no man, ought to be made to choose between forfeiting her position, however high, and fulfilling her wish to serve her country in the Territorial Nursing Service. As the event has proved, had the alternative plan been followed of registering the names of nurses who expressed a wish to serve, and the names of all the training schools and other institutions employing nurses which were willing to co-operate in the event of war to secure the necessary nursing staff, an army of trained nurses, with full and up-to-date knowledge of their work and the highest qualifications in a physical and moral sense, might have been forthcoming, and the selection and appointment of the staff for each Territorial hospital could then have proceeded and been carried out on lines which make for wisdom in the choice and the maximum of efficiency in the Service.

These considerations, though it is necessary to state them plainly in this place, whilst they have added to the difficulties of organisation, have largely ceased to be material owing to the devotion and ability of the matrons as a whole who have been responsible for the executive details and initial administration of each Territorial hospital. We take it that the problems which have given most trouble in their solution may be mainly traceable to the initial plan adopted on the institution of the Service. It has necessarily followed that amongst the nurses who joined the Service originally were many who devoted themselves to private nursing, some of whom have continued in this branch of the work, whilst others have drifted into private work from the idea that it would yield them, on the whole, the best remuneration with the maximum of freedom. Directly war was declared those private nurses who were attached to the Territorials were called upon for active service. Many of them had lived for years outside an institution, with its discipline, order, and regular hours, and not a few of them may have felt it to be irksome to become attached to a Territorial hospital where, in accordance with the regulations, they had, for instance, to be on duty every morning by seven o'clock. Again, others of them had been trained for a number of years, and though working independently, they considered, for that very reason, that they were entitled to be given at once a responsible position and to have a first claim for selection to the office of sister, to which title they attached supreme importance. In fact, the daily life and work of a private nurse does not tend to fit her especially, if at all, to fulfil satisfactorily the responsible office of a sister, and this is particularly the case where a private nurse has left her hospital for so long as to cause her to lose touch with modern conditions of treatment and many other matters with which a sister must be completely familiar.

There are other aspects which affected a considerable number of the original recruits for the Territorial Service which we might mention, but we have said enough to indicate the type of difficulties which have had to be faced by the matrons and overcome. We think it is matter for regret that, although the regulations for the nursing staff for the Territorial hospital system have been drawn up, approved, and issued by the War Office through the Matron-in-Chief of the Service, in some Territorial hospitals, where the matrons have proved to be weak and inferentially unfitted continuously to maintain administrative efficiency, there have been departures, we understand, from the rules for the nurses which ought never to have been permitted; nor would they, we are confident, be allowed if the Matron-in-Chief knew of the breaches in question. Those breaches have included, for instance, we are informed, attempts to satisfy nurses who objected to begin their duties at 7 a.m., and who have been allowed to enter upon them at a later hour. The experience of the hospitals which are most efficiently administered is that a breach of this kind is so serious in practice as to tend to let down and weaken the discipline of the whole establishment.

Again, in regard to the appointment of sisters, where great care has been taken that the first consideration should be the selection of the most competent, and it has been pointed out to the nurses who have been disappointed that they have joined the Service in discharge of their patriotic duty, and that the first duty is to serve in the positions allotted to them in accordance with their fitness for service, a cheerful acceptance has resulted. We may take it, therefore, that where and when resulting jealousies amongst the nurses in regard to the office of sister prevail, it may probably and fairly be attributed to, and taken as evidence of, a weakened enforcement of discipline by the matron as the chief nursing authority in each hospital.

### The Accommodation for the Nurses.

The character of this accommodation seems to vary considerably in quality and quantity, as those who study the block plans of various Territorial hospitals, which we publish elsewhere, may conclude. The truth appears to be that the plans have been settled and agreed to in the absence of a uniform standard of comparison which had been carefully thought out, and provided adequately for the requirements of each department of a temporary hospital upon the best modern lines. The accommodation at the 5th Northern General Hospital at Leicester, the old vacant asylum with temporary additions, with the exception of the situation of the nurses' cloak-room and lavatory, is satisfactory. These temporary quarters reflect credit on the architect, who has provided each nurse with a separate bedroom—a great matter. The whole of these buildings, being temporary in character, have been erected at a notably small expenditure. For this reason they are worthy of study by those interested and others. In addition to the nurses' quarters they include an excellent dining-room, and it is the rule of this hospital that there sisters, nurses, and in fact everybody meet on equal terms.

### The Nurses' Mess.

Experience has shown that the cook-orderlies who are responsible for the food supplied to the patients and staff complain that they find it difficult to cater satisfactorily for the nursing staff. At the 5th Northern General Hospital an excellent system has been established to meet this difficulty. A separate kitchen has been provided for the nurses, which is under the management and control of a sister. A mess fund has been created to which every member of the nursing staff contributes her quota at the rate of so much per week. Each member of the staff has a rations allowance, and of that six-sevenths is contributed to the mess fund, the remaining seventh being kept in reserve as a fund for extras. The members are paid once a month, and at once hand over their rations contribution to the sister. The mess sister selects, orders, and pays for all the provisions, is responsible for the menu, the cooking and the service, and keeps the accounts. This arrangement has given supreme satisfaction to everybody, and the mess sister is so expert and thorough that she often manages to show a surplus on the monthly accounts of the mess, which surplus is divided *pro rata* as a bonus amongst the members. The mess is supplied with every requisite, the table equipment being both good and appetising. We shall be glad to have full particulars of other messes for nurses at Territorial hospitals and elsewhere, to hear in each case something of the methods of supply and the general results attained so far as their nursing establishments are concerned, and to learn which have, and which have not, a separate kitchen. The arrangements, at any rate at the 5th Northern General Hospital, reflect the greatest credit upon everybody concerned.

### The Territorial Cape.

We had an opportunity of going carefully into the question of this cape, which is now made of the same material as the washing dresses. The reasons which influenced Miss Nightingale, having regard to the special circumstances of military, which now include Territorial hospitals, were sound and good. The first consideration with Miss Nightingale was always a high standard of character and self-respect, and she inculcated the spirit of service as a dominant power and influence for every one of her nurses. The cape when well made may be distinctive and attractive. It has one defect due to the Regent Street firm who, we understand, is responsible for the make of these capes. It would appear that the neck portion is not properly cut, for the front of the cape, especially on the right side, often bulges and gives an impression of bad workmanship. It is in practice desirable that the cape should be always worn when the nurses are on duty, for these capes are not in the way and are a protection against cold and chills.

## Figures and Tables

**Figure f1:**